# Precision nitrogen and water management in double zero -till wheat: effects on photosynthetic parameters, productivity, nutrient-use efficiency and N_2_O emission

**DOI:** 10.3389/fpls.2025.1654933

**Published:** 2025-11-07

**Authors:** Vijay Pratap, Anchal Dass, P. Krishnan, S. Sudhishri, Anil K. Choudhary, Arti Bhatia, Dinesh Jinger, Sunil K. Verma, Arjun Singh, Aye Aye San, K. Nithinkumar, K. S. Sachin, Kavita Kumari, R. Sadhukhan, Sandeep Kumar, Venkatesh Paramesha, Teekam Singh, Ramanjit Kaur, Shiv Poojan Yadav

**Affiliations:** 1 Division of Agronomy, ICAR-Indian Agricultural Research Institute, New Delhi, India; 2 R.B.S. College, Bichpuri, Agra, Uttar Pradesh, India; 3 Division of Agricultural Physics, ICAR-Indian Agricultural Research Institute, New Delhi, India; 4 Water Technology Centre, ICAR-Indian Agricultural Research Institute, New Delhi, India; 5 ICAR-Central Potato Research Institute, Shimla, Himachal Pradesh, India; 6 Division of Environmental Science, ICAR-Indian Agricultural Research Institute, New Delhi, India; 7 ICAR-Indian Institute of Soil and Water Conservation, Research Centre-Vasad, Anand, Gujarat, India; 8 Department of Agronomy, Institute of Agricultural Sciences (IAS), Banaras Hindu University (BHU), Varanasi, Uttar Pradesh, India; 9 Department of Agricultural Research, Regional Research Center, Aung Ban, Myanmar; 10 ICAR-Central Rice Research Institute, Cuttack, Odisha, India; 11 Multi-Technology Testing Centre and Vocational Training Centre and College of Horticulture, Central Agricultural University, Imphal, Mizoram, India; 12 ICAR-National Bureau of Soil Survey and Landuse Planning, Regional Centre, Jorhat, Assam, India; 13 ICAR-Central Coastal Agricultural Research Institute, Old Goa, Goa, India; 14 Krishi Vigyan Kendra (KVK), Maharajganj, Uttar Pradesh, India

**Keywords:** crop establishment, nutrient expert, sensors, wheat, photosynthetic parameters, N_2_O emission

## Abstract

**Context:**

Conventional tillage (CT), excessive irrigation, and indiscriminate nitrogen (N) use in wheat farming degrade soil and water resources in the Indo-Gangetic Plains (IGP), threatening the sustainability of the rice-wheat cropping system.

**Objectives:**

A two-year study (2019–21) in north-west IGP was conducted to assess the integration of zero-tillage (ZT) with precision water and N management for sustainability, nutrient efficiency, and environmental performance.

**Methods:**

The study tested two crop establishment methods (ZT-wheat and double ZT-wheat) and three irrigation regimes–25%, 50%, and 75% depletion of available soil moisture (DASM), with silicon applied at 75% DASM–alongside three N strategies: 100% recommended N dose (RDN), NutrientExpert^®^ (NE^®^) + Leaf Color Chart (LCC), and NE^®^ + SPAD-based N management, using a split-plot design.

**Results and Conclusion:**

Double ZT-wheat performed better over conventional ZT, showed superior growth (higher dry matter accumulation, leaf area index, and photosynthetic rate), 3.5% greater interception of photosynthetically active radiation (PAR), and 6.7–9.9% increases in grain/straw yields, and resource-use efficiency. Irrigation at 25% DASM increased photosynthetic activity, intercepted 18.3% more PAR, and yielded 9.23% higher grain over 50% DASM, though delaying irrigation to 50% DASM conserved water without significant yield loss. NE^®^ + SPAD-based N management saved 40 kg N ha^–1^ while enhancing productivity and efficiency, and combining ZT with 75% DASM + silicon and NE^®^ + LCC significantly reduced N_2_O emissions, thus suggested for implementation in the wheat growing regions.

**Significance:**

The current study findings promote precision N-water strategies, and double ZT to enhance productivity, resource conservation, and environmental sustainability in the IGP’s wheat systems addressing important sustainable development goals concerning agriculture.

## Graphical Abstract

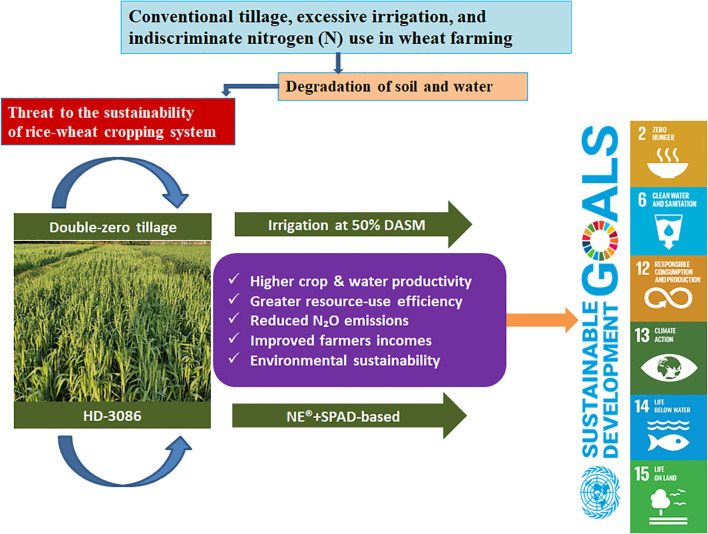

## Highlights

- The study assesses DSS and sensor-guided N supply in wheat in the IGP region.
- NE^®^ + SPAD/LCC-based N supply saved 40 kg N ha^-1^.
- NE^®^ + LCC-based N + ZT and 75% DASM + Si and decreased N_2_O emissions.
- Double ZT-wheat outperformed conventional ZT for photosynthetic parameters and yield.
- Irrigation at 25% DASM was better, but under limited water supply 50% DASM can be ideal schedule.
- The optimal combination was NE+SPAD/LCC based N + double ZT + 25% DASM.

## 1 Introduction

The Indo-Gangetic Plains (IGP) of South Asia, a cornerstone of India’s food security (Rajanna et al., 2023), have relied on intensive tillage-based rice-wheat systems, supplying 54% of rice and 84% of wheat to the nation’s public distribution system (Fagodiya et al., 2023). Practices like intensive tillage, unchecked irrigation, and disproportionate fertilizer use degrade soil health, deplete groundwater, and harm ecosystems and human well-being (Jinger et al., 2023a). Resource-efficient crop establishment techniques and precision water and nitrogen (N) management are imperative for sustaining India’s agrarian future (Yadav et al., 2021; Kumar et al., 2022; Rajanna et al., 2023). Conservation agriculture (CA) is an eco-friendly farming practice that has proven especially effective for cereal crops in the north-west India, where it boosts soil fertility and crop yields (Kumar et al., 2021; Jat et al., 2021). Retaining crop residues helps buffer crops against heat and drought stress (Dass and Bhattacharyya, 2017) by enriching soil organic carbon, improving moisture retention, and moderating soil temperature (Pratap et al, 2023; Jat et al., 2025). Beyond boosting farm output, zero-till practices offer economic, ecological, and societal advantages, positioning them as vital tools to combat climate change, soil degradation, and escalating input cost (Verma and Singh, 2009; Keil et al., 2020). Studies highlight that CA-based tillage and crop management not only enhance short-term yields but also improve soil quality and agricultural sustainability (Kumar et al., 2021).

Research from the IGPs highlights imbalances in nutrient management practices: farmers tend to over apply N, under apply phosphorus (P) and inadequately address potassium (K), sulfur (S), and micronutrient requirements (Sapkota et al., 2021a; Vijayakumar et al., 2021; Jinger et al., 2023b). These practices emphasize systemic inefficiencies in fertilization strategies, potentially compromising long-term soil fertility and crop sustainability. The high variability in soil N availability reduces the efficiency of blanket fertilizer recommendations, resulting in imbalanced N application (Dobermann et al., 2002). This disparity between crop N requirements and fertilizer application, combined with excessive fertilization, causes substantial environmental damage (Cui et al., 2018). Such practices lead to several consequences, including diminished farm profitability, low nutrient-use efficiency, intensified climate change impacts, and broader ecological degradation (Arunachalam et al., 2025). Recent innovations, such as Site-Specific Nutrient Management (SSNM) and precision agriculture tools enable dynamic, real-time N management. Conventional blanket fertilizer recommendations fail to capture field variability, leading to nutrient inefficiency and environmental losses (Cui et al., 2018). Precision tools such as NutrientExpert^®^ (NE), leaf color charts (LCCs), GreenSeeker, and SPAD meters provide site-specific solutions that align crop demand with nutrient and water supply (Pratap et al., 2022). NE, a computer-based decision support tool, generates fertilizer recommendations based on soil, crop, and management conditions, improving N-use efficiency (NUE) and profitability while lowering N_2_O emissions (Sapkota et al., 2021a). LCCs offer a low-cost method for farmers to fine-tune N application through leaf greenness monitoring. Optical sensors like GreenSeeker and SPAD meters provide real-time assessment of crop vigor and chlorophyll content, guiding dynamic adjustments in N and irrigation management (Jat et al., 2022). Integrating these tools with soil moisture-based irrigation scheduling has been shown to increase yield, reduce fertilizer use, and enhance water productivity (Gupta et al., 2023). Overall, these innovations outperform conventional practices by optimizing inputs, improving resource-use efficiency, and reducing environmental footprints, thereby advancing climate-smart wheat production systems in South Asia. Rising water scarcity, exacerbated by climate change, poses significant challenges in enhancing food production (UNWWDR, 2018). Concurrently, water availability is declining globally due to rapid population growth, urbanization, industrial expansion, and climate-related disruptions (Du Plessis et al., 2019). Irrigation, accounts for only 19% of agricultural land and provides 40% of world food, and improved farm income (Hanjra et al., 2009). However, over the decades, farmers have irrigated wheat during critical growth stages, which requires a huge quantity of water. Thus, precise application of irrigation using soil moisture sensors like FDR (frequency domain reflectometry) for determining the time and volume of irrigation seems as a vital strategy towards sustainable cereal-based agricultural productions (Kumar et al., 2025). Measuring irrigation water using electronic devices or sensors like star-flow meters further improves precision in irrigation (Behera and Sharma, 2014; Gupta et al., 2023). Apart from this, deficit irrigation with stress-alleviating materials, such as silicon (Si) would be a practicable tactic to save water while concurrently obtaining reasonable productivity (Salem et al., 2021a; Jinger et al., 2020). Silicon application under water-limited conditions enhances a crop’s drought resilience by stabilizing plant hydration, sustaining photosynthetic activity, and preserving leaf structure and xylem integrity under elevated temperatures and moisture deficits (Pratap et al., 2022), ultimately contributing to improved grain productivity (Jinger et al., 2021). Although several studies have independently investigated the effects of dual zero-tillage (DZT) on crop productivity and resource-use efficiency, as well as the role of precision nitrogen and water management practices in improving system sustainability, these studies have largely remained fragmented.

As N availability, absorption, translocation, and assimilation in crops are highly influenced by soil moisture and tillage conditions (Liang et al., 2019a), the conventional way of uniform fertilizer scheduled often fails to address field heterogeneity, leading to inefficiencies and land degradation. Precision N management tools, such as NE, GreenSeeker, and SPAD meter, optimize N-use based on site-specific conditions, but their effectiveness under varied tillage and moisture conditions is still largely unexplored. To date, there is a lack of systematic field-based evidence assessing the combined effects of DZT × precision nitrogen × precision irrigation in the Indo-Gangetic Plains. This knowledge gap underlines the need for integrated evaluations to understand potential synergies, thereby providing the basis for our present investigation. We hypothesize that the integration of these precision tools into N management strategies under diverse tillage (ZT/DZT) and moisture conditions will enhance nutrient-use efficiency, improve crop productivity, and minimize environmental footprints. Additionally, we suggest that real-time data from these tools will allow for timely and accurate interventions, thereby contributing to sustainable intensification. This study aimed to examine the impact of (1) precision N and water management strategies on crop growth, photosynthetic activity, and the interception of photosynthetically active radiation in ZT/DZT wheat, and (2) effective N and water management practices on ZT/DZT wheat productivity, resource-use efficiency, and N_2_O emissions.

## 2 Materials and methods

### 2.1 Experimental site

**Figure 1:**
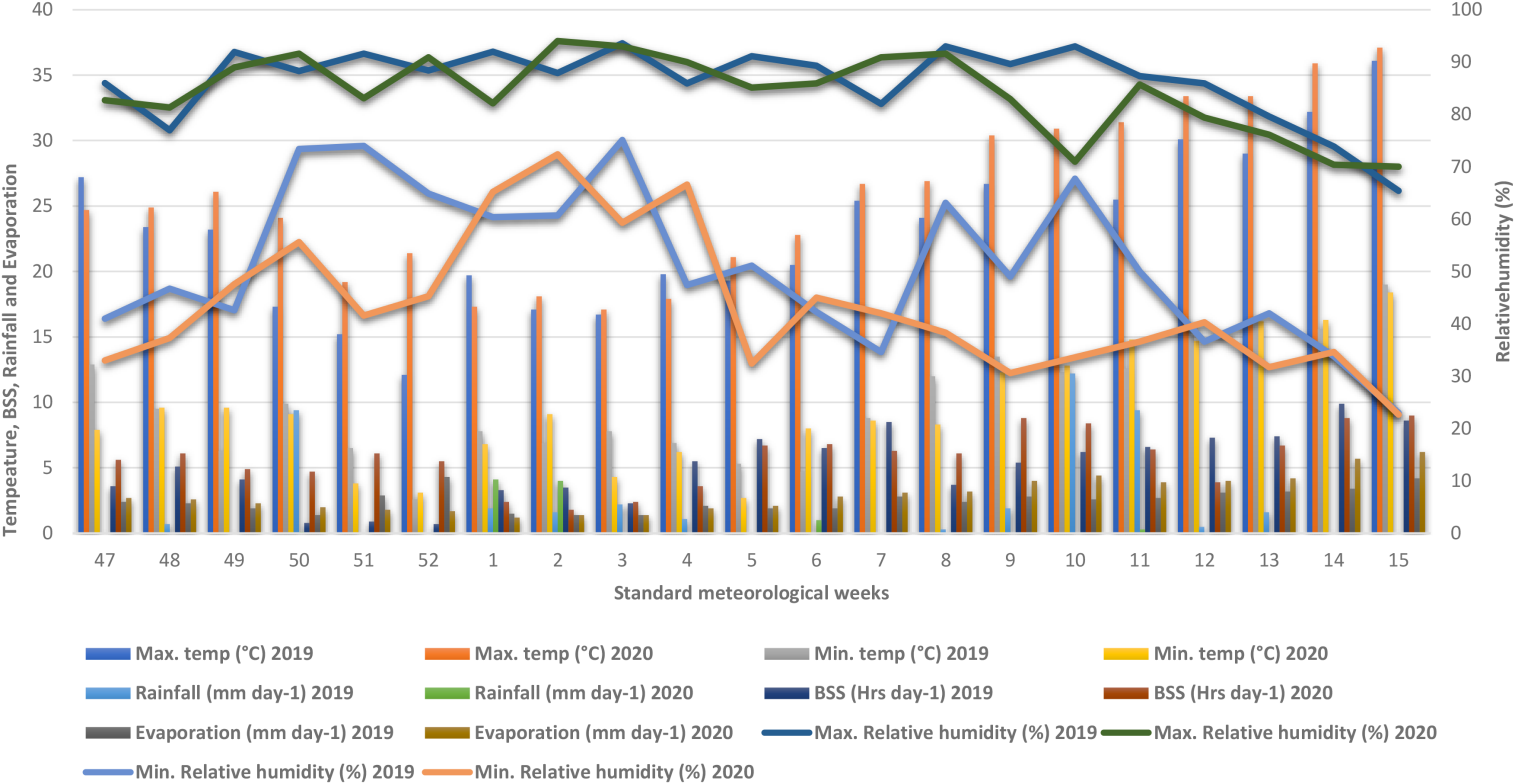
Weekly meteorological data of winter season during 2019–20 and 2020–21.

The field experiment was conducted over two consecutive winter seasons (November to mid-April) in 2019–20 and 2020–21 at the ICAR–Indian Agricultural Research Institute, New Delhi, India (28°38’N, 77°09’E; 229 m above mean sea level), a region characterized by a sub-tropical, semi-arid climate with hot, dry summers and cold winters (Dass and Bhattacharyya, 2017). During the study periods, the mean maximum temperatures were 39.4°C (2019–20) and 40°C (2020–21), while the mean minimum temperatures dropped to 0.6°C and -0.8°C, respectively, with total seasonal rainfall recorded at 299.5 mm (2019–20) and 65.9 mm (2020–21) (**Figure 1**). The experimental soil, a sandy clay loam classified as Typic Haplustept, exhibited a pH of 8.3 (1:2.5 soil:water suspension; Piper, 1950), soil organic carbon (SOC) content of 0.41% (Walkley and Black, 1934), available N of 176.2 kg ha^−1^(alkaline KMnO_4_ oxidizable; Subbiah and Asija, 1956), available P of 11.6 kg ha^−1^(0.5 M NaHCO_3_ extractable; Olsen et al., 1954), and available potassium (K) of 272.5 kg ha^−1^(1 N NH_4_OAc extractable; Hanway and Heidel, 1952).

### 2.2 Experimental design

The field experiment was conducted in a thrice replicated split-plot design (Rangaswamy, 2018). The main-plots tested two crop establishment methods: zero-tilled wheat (ZT wheat) and double zero-tilled (DZT) wheat (involving zero-till direct-seeded rice followed by ZT wheat), and three irrigation regimes: irrigation at 25% depletion of available soil moisture (DASM), 50% DASM, and 75% DASM supplemented with Si at 80 kg ha^−1^. Sub-plots were allocated to three N management strategies: 100% of the recommended N rate (150 kg ha^−1^), Nutrient Expert^®^ combined with a leaf color chart (LCC), and Nutrient Expert^®^ paired with a SPAD meter for precision N application (Pratap et al., 2022). Treatment details have been mentioned in the **Table 1**. Rice residue from the prior season was uniformly applied at 3.5 t ha^−1^across all treatments.

**Table 1:** Treatment details.

Main-plot	
A. Crop establishment	
1.	Zero-tilled wheat
2.	Double zero-tilled wheat (zero-till direct-seeded rice followed by ZT wheat)
B. Irrigation regimes	
1.	25% Depletion of available soil moisture (DASM)
2.	50% DASM
3.	75% DASM + Si (80 kg ha^-1^)
C. Sub-plot: Precision N management	
1.	Recommended N rate (150 kg ha^-1^)
2.	Nutrient Expert + LCC
3.	Nutrient Expert + SPAD meter

### 2.3 Crop management

One week before wheat sowing, glyphosate was uniformly applied at 1.0 kg ai ha^-1^ to control weeds. The high-yielding wheat cultivar ‘HD 3086’ (143-day maturity period), was sown at a rate of 100 kg of seed per hectare utilizing a ferti-cum-seed drill with 22.5 cm row spacing on November 21 and November 18 during the first and second study years, respectively. On the sowing day, pendimethalin (a pre-emergence herbicide) was manually sprayed at 1000 g ai ha^–1^. The recommended nitrogen dose (RDN) was applied at 150 kg ha^-1^, while precision N management utilized the NE^®^ tool to calculate site-specific N requirements. The NE^®^ software determined the required nutrient application rates as 110 kg N, 42 kg P_2_O_5_, and 40 kg K_2_O ha^-1^. During sowing, one-third of the N and the entire doses of P_2_O_5_ and K_2_O were applied as a basal fertilizer, using urea, single super phosphate, and muriate of potash as nutrient sources. The remaining N was top-dressed later in the season, triggered when leaf color chart (LCC) readings fell below 4 or SPAD meter values dropped ≤44. Additionally, Si (80 kg ha^−1^), supplied as calcium silicate, was incorporated at sowing to enhance the crop’s resilience to water stress. Treatment-wise post-establishment irrigation schedules are shown in **Table 2**.

**Table 2:** Details of irrigation schedules in wheat.

Irrigation regimes	No. of irrigation		Depth of irrigation water (mm)	Total applied irrigation water (mm)	
	ZT- wheat	ZT-wheat	ZT-wheat	ZT-wheat	ZT-wheat
	2019-20	2020-21	2019-20 & 2020-21	2019-20	2020-21
Irrigation at 25% DASM	4	5	50	200	250
Irrigation at 50% DASM	3	4	55	165	220
Irrigation at 75% DASM+Si_80_	2	3	60	120	180

Layer-wise soil moisture content was measured using a soil profile moisture sensor (FDR). The volume of irrigation water was measured using a water flow meter installed at the outlet of a cement-concrete channel connecting the experimental plot. To keep the weed population below the threshold level, sulfosulfuron + metsulfuron was applied at a rate of 30 + 2 g a.i. ha^−1^, 35 days after sowing (DAS). This was followed by hand-weeding 50 DAS to eliminate any late-emerging weeds.

### 2.4 Data collection

#### 2.4.1 Growth parameters

To assess dry matter accumulation (DMA), two randomly selected spots, each measuring 50 cm × 50 cm, were marked. Plant samples were collected, then sun-dried and subsequently oven-dried at 70 ± 2°C until a constant weight was reached. Leaf area was measured electronically using a leaf area meter (Model LICOR 3100, LICOR Inc., Lincoln, USA). The leaf area index (LAI) was calculated using the following Equation 1 (Evans, 1972):

(1) $$\text{LAI}=\frac{\text{Total leaf area }({\text{cm}}^{2})}{\text{Ground area }({\text{cm}}^{2})}$$

#### 2.4.2 SPAD and NDVI values

Data on SPAD value was recorded using a hand-held chlorophyll meter (Minolta SPAD-502). The normalized difference vegetation index (NDVI) was measured using an optical sensor (a hand-held GreenSeeker) by moving it 0.5 m above crop canopy.

#### 2.4.3 Photosynthetic behavior

On a bright sunny day, net photosynthetic rate (NPR) of top-most fully opened leaf of healthy plant was measured at two places in each plot using an infrared gas analyzer (LI-COR, model LI-6400XT Portable Photosynthesis System) at active tillering and flowering stages. Data on stomatal conductance and transpiration rate were also recorded while measuring NPR. Transpiration efficiency was calculated using the following Equation 2 (Sinclair and Muchow, 2001):

(2) $$\text{Transpiration efficiency}=\frac{\text{Net photosynthetic rate }({\text{ µ mol CO}}_{2}{\text{m}}^{-2\;}{\text{s}}^{-1})}{\text{Transpiration rate }({\text{m mol H}}_{2}{\text{O m}}^{-2}{\text{ s}}^{-1})}$$

#### 2.4.4 Photosynthetically active radiation interception

At crop canopy, total incident radiation and transmitted PAR were measured by randomly placing canopy analyser LP-80 AccuPAR in between the crop rows at two spots in each plot. Net intercepted PAR by crop was computed by subtracting value of transmitted PAR (at bottom of crop) from total incident radiation at top of the canopy. Intercepted PAR (%) was computed as (Intercepted PAR/Total incident PAR) ×100).

#### 2.4.5 Yield attributes and yields

The yield attributing characters like effective tillers, grains spike^-1^ and test-weight (g) were determined. After discarding three crop rows from all sides as border, produce of each plot was harvested manually, sun-dried, tagged and weighed with a portable swing balance in order to obtain biological yield and expressed in tons. The harvested material was threshed using a Pullman thresher, grains cleaned, sun-dried for 4–5 days, and weighed to determine grain yield, with straw yield calculated as the difference between biological and grain yields.

#### 2.4.6 Partial factor productivity of N, P, and K

Partial factor productivity of N, P, and K was calculated using the Equation 3 (Dobermann, 2007):

(3) $$\text{Partial factor productivity }(\text{PFP})=\frac{\text{Grain yield }\left({\text{kg ha}}^{-1}\right)}{\text{Amount of nutrient applied}\;({\text{kg ha}}^{-1})}$$

#### 2.4.7 Irrigation water productivity

Irrigation water productivity (IWP) was calculated using the following Equation 4 (Zwart and Bastiaanssen, 2004):

(4) $$\text{Irrigation water productivity}=\frac{\text{Grain yield }({\text{kg ha}}^{-1})}{\text{Irrigation water used }(\text{mm})}$$

#### 2.4.8 N_2_O emission

The study investigated N_2_O emissions in wheat using a closed chamber technique adapted from Debnath et al. (1996). After crop sowing, iron channels were inserted 10 cm into the soil to enclose 2–3 crop rows, and acrylic chambers (30 cm × 30 cm × 100 cm) were mounted on these channels during gas sampling, with water sealing the base to ensure airtight conditions. Gas samples were collected at 0- and 1-hour intervals using a 50 mL syringe fitted with a 24-gauge needle through a silicon septum on the chamber, while a battery-operated pump homogenized internal air. Post-collection, syringes were sealed with a three-way stopcock, and air temperature and chamber headspace volume were recorded. Sampling occurred during fertilizer and irrigation events, spanning three days per cycle across the season. N_2_O-N concentrations were analyzed using a Gas Chromatograph (Hewlett Packard 5890 Series II) equipped with an electron capture detector and a Porapak N column, maintained at 50°C (column), 120°C (injector), and 320°C (detector), with N carrier gas flowing at 14 mL min^-1^.

#### 2.4.9 Analysis of N_2_O

A gas chromatograph (GC) fitted with an electron capture detector (ECD) and the concentration of nitrous oxide in the gas samples was measured using a 6’×1/8″stainless steel column (Porapak N). Electrophilic substance detection was carried out using the ECD. The detector consists of two electrodes, one of which is radioactively treated with 63-Ni (or titanium or scandium) to generate beta radiation. High energy electrons bombard the carrier gas (N_2_ or argon mixture), resulting in the production of numerous low energy secondary electrons. These electrons are collected by the positively polarized electrode on the other end.

Electrons captured by an electrophilic sample component passing through the electrode gap, resulting in electrical reproduction of the GC peak, decreases the steady state current. The temperature of the column and detector was kept at 50°C and 300°C, respectively. The carrier back flush and detector purge gases (which are composed of 95% argon and 5% methane or N_2_) were maintained at 14–18 cm^3^ minute^-1^. Gas injection ports were used to introduce gas samples into at gas sampling loop. The gas samples were cleaned of both CO_2_ and water vapor. Two absorbent traps were created using10-mm milli pore syringe filter holders filled with Ascarite and MgClO_4_. The peak area was plotted and measured using GC software. Primary standards are the N_2_O standard (300, 500, and 1000 ppbV). The following formula was used to determine the nitrous oxide flux:

Cross-sectional area of the chamber (m^2^) = A

Headspace (m) = H

Volume of head space (L) = 1000 × AH,

N_2_O concentration at 0 time (μL/L) = Co,

N_2_O concentration after time t (μL/L) = Ct,

Change in concentration in time t(μL/L) = (Ct-Co),

Volume of N_2_O evolved in time t (μL) = (Ct-Co) × 1000AH

When t is in hours, then flux (mL/m^2^/h^-1^) = [(Ct-Co) × AH)/(A× t)

Now 22.4 mL of N_2_O is 44 mg at standard temperature and pressure,

Hence, N_2_O flux = [(Ct-Co)/t] × H × 44/22.4 × 10000 × 24 mg ha^-1^ day^-1^

#### 2.4.10 Statistical analysis

Data were analyzed using ANOVA (Gomez and Gomez, 1984), with treatment significance tested by the F-test (p ≤ 0.05). Duncan’s multiple range test (DMRT) compared means, and analyses were performed using SAS 9.3 (SAS Inst. Inc., Cary, NC, USA).

## 3 Results

### 3.1 Growth parameters

In a comparative study of wheat treatments, DZT-wheat demonstrated superior performance, achieving higher LAI at the flowering stage and dry matter accumulation (DMA) at harvest than conventional ZT-wheat. Scheduling irrigation at 25% depletion of available soil moisture (DASM) led to significantly higher LAI over 75% DASM + Si_80_ treatment, while irrigation at 50% DASM showed statistically comparable results to 25% DASM. Additionally, 25% DASM irrigation enhanced DMA by 4.6% and 4.0% over 50% DASM, and by 45.1% and 33.8% over 75% DASM + Si_80_ during 2019–20 and 2020–21, respectively. The NE+SPAD meter approach outperformed RDN, registering higher LAI and DMA though results were statistically similar to the NE+LCC method across both seasons (**Table 3**).

**Table 3:** Effect of crop establishment methods, irrigation regimes and precision N management options on dry matter accumulation, LAI, and NDVI of wheat.

Treatments	Dry matter accumulation (g m^-2^) at harvest		Leaf area index at flowering stage		NDVI value			
					Maximum tillering stage		Flowering stage	
	2019-20	2020-21	2019-20	2020-21	2019-20	2020-21	2019-20	2020-21
Crop establishment methods								
Zero till-wheat	1035.3^b^	1169.4^b^	4.38^b^	4.75^b^	0.49^a^	0.51^a^	0.47^a^	0.60^a^
Double zero till-wheat	1105.2^a^	1224.2^a^	4.54^a^	4.86^a^	0.51^a^	0.53^a^	0.49^a^	0.62^a^
SEm±	14.30	17.01	0.03	0.03	0.01	0.01	0.01	0.01
LSD (P = 0.05)	45.00	53.52	0.10	0.10	NS	NS	NS	NS
Irrigation regimes								
Irrigation at 25% DASM	1213.7^a^	1325.4^a^	4.65^a^	5.02^a^	0.55^a^	0.56^a^	0.54^a^	0.65^a^
Irrigation at 50% DASM	1160.3^a^	1274.5^a^	4.57^a^	4.90^a^	0.52^a^	0.53^b^	0.50^b^	0.62^b^
Irrigation at 75% DASM + Si (80 kg ha^-1^)	836.7^b^	990.4^b^	4.17^b^	4.50^b^	0.43^b^	0.46^c^	0.41^c^	0.55^c^
SEm±	17.52	20.83	0.04	0.04	0.01	0.01	0.01	0.01
LSD (P = 0.05)	55.12	65.55	0.13	0.12	0.03	0.02	0.02	0.03
N management options								
Recommended N rate	1039.6^b^	1163.6^b^	4.30^b^	4.66^b^	0.48^b^	0.50^b^	0.47^b^	0.59^b^
Nutrient Expert +LCC	1074.5^ab^	1206.2^a^	4.51^a^	4.86^a^	0.50^a^	0.52^a^	0.49^a^	0.61^ab^
Nutrient Expert + SPAD meter	1096.6^a^	1220.5^a^	4.58^a^	4.90^a^	0.52^a^	0.53^a^	0.50^a^	0.62^a^
SEm±	15.18	15.07	0.04	0.04	0.01	0.01	0.01	0.01
LSD (P = 0.05)	44.31	44.00	0.11	0.11	0.03	0.01	0.03	0.02

Treatment means with similar superscripted letters within a column are not significantly different at P < 0.05 according to Tukey’s honestly significant difference test.

### 3.2 NDVI values

The highest NDVI values at the maximum tillering stage and flowering stages were recorded with DZT-wheat, and the lowest with ZT-wheat during both years. Irrigation at 25% DASM recorded higher NDVI values at maximum tillering, which were significantly higher than irrigation at 75% DASM + Si_80_. Similarly, at the flowering stage, the highest NDVI values were found with irrigation at 25% DASM, followed by 50% DASM and 75% DASM + Si_80_. Among precision N-management options, NE+ SPAD meter at maximum tillering was significantly superior to RDN, and stood alike NE+ LCC. At the flowering stage, the highest NDVI values were found with NE+ SPAD and the lowest with RDN (**Table 3**).

### 3.3 Photosynthetic behavior

In a two-year study evaluating physiological responses in wheat, DZT wheat exhibited higher NPR (18.2 and 19.9 µmol CO_2_ m^−2^ s^−1^), stomatal conductance (0.76 and 0.84 µmol CO_2_ m^−2^ s^−1^), and transpiration rates (3.0 and 3.1 mmol H_2_O m^−2^ s^−1^) at maximum tillering compared to conventional ZT wheat. However, ZT wheat demonstrated greater photosynthetic efficiency (7.58 and 7.37 µmol CO_2_ [mmol H_2_O]^−1^), a trend consistent at the flowering stage. Applying irrigation at 25% depletion of available soil moisture (DASM) achieved the highest NPR (19.1 and 20.9 µmol CO_2_ m^−2^ s^−1^), stomatal conductance (0.79 and 0.88 µmol CO_2_ m^−2^ s^−1^), and transpiration (3.3 and 3.4 mmol H_2_O m^−2^ s^−1^), closely followed by 50% DASM and 75% DASM + Si_80_. Despite this, 75% DASM + Si_80_ showed superior photosynthetic efficiency (5.79 and 6.15 µmol CO_2_ [mmol H_2_O]^−1^), outperforming other irrigation levels. In nitrogen management, the NE+SPAD meter approach yielded the highest NPR (18.3 and 20.1 µmol CO_2_ m^−2^ s^−1^), stomatal conductance (0.76 and 0.84 µmol CO_2_ m^−2^ s^−1^), and transpiration (2.9 and 3.1 mmol H_2_O m^−2^ s^−1^), significantly exceeding RDN, while NE+LCC results were statistically comparable to NE+SPAD. Conversely, RDN recorded the highest photosynthetic efficiency (7.04 and 7.08 µmol CO_2_ [mmol H_2_O]^−1^), with NE+SPAD showing the lowest values, a pattern mirrored at the flowering stage across both study years (**Tables 4**, **5**).

**Table 4:** Effect of crop establishment methods, irrigation regimes and precision N management options on photosynthetic parameters at maximum tillering stage of wheat.

Treatments	Photosynthetic rate (µ mol CO_2_ m^-2^ s^-1^)		Stomal conductance (µ mol CO_2_ m^-2^ s^-1^)		Transpiration rate (m mol H_2_O m^-2^ s^-1^)		Photosynthetic efficiency (μ mol CO_2_ (m mol H_2_O)^-1^)	
	2019-20	2020-21	2019-20	2020-21	2019-20	2020-21	2019-20	2020-21
Crop establishment methods								
Zero till-wheat	17.2^b^	18.8^b^	0.67^b^	0.75^b^	2.40^b^	2.70^b^	7.58^b^	7.37^b^
Double zero till-wheat	18.2^a^	19.9^a^	0.76^a^	0.84^a^	3.01^a^	3.10^a^	5.73^a^	6.07^a^
SEm±	0.28	0.18	0.02	0.02	0.08	0.11	–	–
LSD (P = 0.05)	0.89	0.55	0.05	0.07	0.25	0.36	–	–
Irrigation regimes								
Irrigation at 25% DASM	19.1^a^	20.9^a^	0.79^a^	0.88^a^	3.26^a^	3.43^a^	5.79^a^	6.15^a^
Irrigation at 50% DASM	18.6^a^	19.9^b^	0.74^a^	0.81^b^	2.82^b^	2.99^a^	6.64^b^	6.63^b^
Irrigation at 75% DASM + Si (80 kg ha^-1^)	15.4^b^	17.2^c^	0.61^b^	0.69^b^	2.04^b^	2.28^b^	7.70^c^	7.54^c^
SEm±	0.35	0.22	0.02	0.03	0.10	0.14	–	–
LSD (P = 0.05)	1.09	0.68	0.06	0.09	0.31	0.44	–	–
N management options								
Recommended N rate	16.9^b^	18.4^b^	0.64^b^	0.73^b^	2.44^b^	2.62^b^	7.04^b^	7.08^b^
Nutrient Expert +LCC	17.8^a^	19.5^a^	0.73^a^	0.82^a^	2.78^a^	3.00^a^	6.36^a^	6.50^a^
Nutrient Expert + SPAD meter	18.3^a^	20.1^a^	0.76^a^	0.84^a^	2.89^a^	3.08^a^	6.31^a^	6.48^a^
SEm±	0.27	0.29	0.02	0.03	0.10	0.13	–	–
LSD (P = 0.05)	0.80	0.83	0.04	0.07	0.30	0.36	–	–

Treatment means with similar superscripted letters within a column are not significantly different at P < 0.05 according to Tukey’s honestly significant difference test.

**Table 5:** Effect of crop establishment methods, irrigation regimes and precision N management options on photosynthetic parameters at flowering stage of wheat.

Treatments	Photosynthetic rate (µ mol CO_2_ m^-2^ s^-1^)		Stomal conductance (µ mol CO_2_ m^-2^ s^-1^)		Transpiration rate (m mol H_2_O m^-,2^ s^-1^)		Photosynthetic efficiency (μ mol CO_2_ (m mol H_2_O)^-1^)	
	2019-20	2020-21	2019-20	2020-21	2019-20	2020-21	2019-20	2020-21
Crop establishment methods								
Zero till-wheat	23.2^b^	24.0^b^	0.41^a^	0.50^a^	5.13^a^	5.36^a^	4.77^b^	4.74^b^
Double zero till-wheat	24.3^a^	25.6^a^	0.45^a^	0.58^a^	4.96^a^	5.28^a^	4.64^a^	4.53^a^
SEm±	0.22	0.27	0.02	0.02	0.09	0.13	–	–
LSD (P = 0.05)	0.69	0.84	NS	0.08	NS	0.41	–	–
Irrigation regimes								
Irrigation at 25% DASM	25.7^a^	26.8^a^	0.51^a^	0.60^a^	5.72^a^	6.03^a^	4.51^a^	4.47^a^
Irrigation at 50% DASM	24.1^b^	25.3^b^	0.45^a^	0.54^a^	5.13^a^	5.37^b^	4.73^b^	4.69^b^
Irrigation at 75% DASM + Si (80 kg ha^-1^)	21.4^c^	22.3^c^	0.33^b^	0.47^b^	4.28^b^	4.56^c^	4.98^c^	4.85^c^
SEm±	0.27	0.33	0.03	0.03	0.11	0.16	–	–
LSD (P = 0.05)	0.85	1.03	0.08	0.09	0.34	0.50	–	–
N management options								
Recommended N rate	23.1^b^	24.0^b^	0.38^b^	0.48^b^	4.78^b^	5.04^b^	4.81^b^	4.80^b^
Nutrient Expert +LCC	24.0^ab^	25.1^ab^	0.44^a^	0.55^a^	5.11^a^	5.41^a^	4.71^a^	4.64^a^
Nutrient Expert + SPAD meter	24.2^a^	25.4^a^	0.46^a^	0.58^a^	5.25^a^	5.52^a^	4.57^a^	4.62^a^
SEm±	0.36	0.36	0.10	0.02	0.10	0.16	–	–
LSD (P = 0.05)	1.06	1.05	0.30	0.05	0.28	0.46	–	–

Treatment means with similar superscripted letters within a column are not significantly different at P < 0.05 according to Tukey’s honestly significant difference test.

### 3.4 Photosynthetic-active radiation interception

In a two-year study assessing PAR interception in wheat, DZT wheat demonstrated superior performance, intercepting 2.4–5.4% more PAR across growth stages compared to conventional ZT wheat (**Table 6**). Wheat irrigated at 25% DASM captured the highest PAR levels at maximum tillering (667.4 and 982.7 µmol CO_2_ m^−2^ s^−1^), significantly exceeding values for 50% DASM and 75% DASM + Si_80_. At flowering, 25% DASM irrigation maintained its dominance for intercepted PAR surpassing 50% DASM by over 5% and 75% DASM + Si_80_ by over 18%. For N management, the NE+SPAD meter approach achieved the highest PAR interception both at maximum tillering and flowering stages, outperforming NE+LCC by 1.3–3.2% and RDN by 3.8–7.5%, underscoring its efficacy in optimizing light capture.

**Table 6:** Effect of crop establishment methods, irrigation regimes and precision N management options on photo synthetically active radiation interception at maximum tillering and flowering stages of wheat.

Treatments	Total incident radiation (µ mol CO_2_ m^-2^ s^-1^) at tillering		Intercepted PAR (µ mol CO_2_ m^-2^ s^-1^) at tillering		Total incident radiation (µ mol CO_2_ m^-2^ s^-1^) at flowering		Intercepted PAR (µ mol CO_2_ m^-2^ s^-1^) at flowering	
	2019-20	2020-21	2019-20	2020-21	2019-20	2020-21	2019-20	2020-21
Crop establishment methods								
Zero tilled-wheat	882.5^b^	1300.0^b^	523.9^b^	822.7^b^	1494.2^a^	1304.6^b^	708.9^a^	725.8^b^
Double zero tilled-wheat	954.7^a^	1432.9^a^	584.7^a^	982.6^a^	1522.6^a^	1453.8^a^	762.0^a^	883.1^a^
SEm±	13.05	14.94	14.65	17.53	14.67	13.57	21.88	17.40
LSD (P = 0.05)	41.05	47.02	46.11	55.17	NS	42.69	NS	53.84
Irrigation regimes								
Irrigation at 25% DASM	1005.2^a^	1421.1^a^	667.4^a^	982.7^a^	1644.9a	1523.4a	925.6a	990.2a
Irrigation at 50% DASM	946.03^b^	1373.1^b^	572.8^b^	908.5^b^	1505.4b	1404.8b	761.9b	829.6b
Irrigation at 75% DASM + Si (80 kg ha^-1^)	804.6^c^	1305.0^c^	422.6^c^	816.8^c^	1374.8c	1209.3c	518.9c	593.6c
SEm±	15.98	18.30	17.95	21.47	17.98	16.62	26.80	21.31
LSD (P = 0.05)	50.28	57.59	56.47	67.57	56.54	52.29	84.34	67.07
N management options								
Recommended N rate	901.5^a^	1330.2^b^	523.4^a^	851.0^a^	1491.0^b^	1314.5^b^	671.1^b^	727.7^b^
Nutrient Expert +LCC	916.2^a^	1370.9^a^	556.2^a^	910.0^a^	1515.6^a^	1397.1^a^	743.0^a^	824.6^a^
Nutrient Expert + SPAD meter	938.2^a^	1398.2^a^	583.3^a^	946.9^a^	1518.6^a^	1426.0^a^	792.3^a^	861.2^a^
SEm±	19.77	14.45	27.26	15.53	25.36	24.22	26.95	26.22
LSD (P = 0.05)	57.71	42.17	NS	45.32	NS	70.69	78.67	76.53

Treatment means with similar superscripted letters within a column are not significantly different at P < 0.05 according to Tukey’s honestly significant difference test.

### 3.5 Yield attributes and yield

Double zero-tillage (DZT) wheat demonstrated consistent improvements over conventional ZT wheat across both study years. Effective tiller counts increased by 5.6% under DZT. Additionally, DZT wheat showed modest enhancements in spike-related parameters, including grains spike^-1^ and 1000-grain weight. Notably, grain yield (4.77–5.48 t ha^−1^) and straw yield (7.51–8.12 t ha^−1^) under DZT exceeded conventional ZT by 7.4–6.0% and 6.37–3.83%, respectively during the first and second study year, respectively (**Table 7**).

**Table 7:** Effect of crop establishment methods, irrigation regimes and precision N management options on yield attributes, yield and irrigation water productivity of wheat.

Treatments	Effective tiller m^-2^		Grains spike^-1^		1000-grain weight		Grain yield (t ha^-1^)		Straw yield (t ha^-1^)		Irrigation water productivity (kg ha^-1^ mm^-1^)	
	2019-20	2020-21	2019-20	2020-21	2019-20	2020-21	2019-20	2020-21	2019-20	2020-21	2019-20	2020-21
Crop establishment methods												
Zero till-wheat	377^a^	385^b^	55.28^a^	59.28^a^	41.0^a^	42.0^a^	4.44^b^	5.17^b^	7.06^b^	7.82^b^	27.6^b^	23.9^b^
Double zero till-wheat	397^a^	408^a^	56.87^a^	61.11^a^	41.3^a^	42.5^a^	4.77^a^	5.48^a^	7.51^a^	8.12^a^	29.8^a^	25.2^a^
SEm±	6.50	6.98	1.40	0.93	0.16	0.15	0.08	0.09	0.13	0.07	0.56	0.41
LSD (P = 0.05)	20.47	21.96	NS	NS	NS	NS	0.26	0.28	0.41	0.23	1.75	1.30
Irrigation regimes												
Irrigation at 25% DASM	428^a^	438^a^	61.06^a^	66.47^a^	42.2^a^	43.4^a^	5.39^a^	6.04^a^	8.56^a^	9.14^a^	26.9^b^	24.0^a^
Irrigation at 50% DASM	390^b^	399^b^	58.14^a^	62.94^a^	41.3^b^	42.4^b^	4.86^b^	5.61^b^	7.57^b^	8.09^b^	29.5^a^	25.5^a^
Irrigation at 75% DASM + Si (80 kg ha^-1^)	343^c^	351^c^	49.03^b^	51.17^b^	40.0^c^	41.0^c^	3.57^c^	4.32^c^	5.73^c^	6.69^c^	29.7^a^	24.2^a^
SEm±	7.97	8.55	1.72	1.14	0.20	0.18	0.10	0.11	0.16	0.09	0.68	0.51
LSD (P = 0.05)	25.07	26.90	5.41	3.57	0.63	0.57	0.32	0.34	0.51	0.28	2.15	1.59
N management options												
Recommended N rate	371^c^	379^c^	52.00^b^	56.39^b^	41.0^c^	42.0^c^	4.47^b^	5.12^b^	7.08^b^	7.81^b^	27.9^b^	23.7^b^
Nutrient Expert + LCC	389^b^	399^b^	56.78^a^	60.81^a^	41.2^b^	42.3^b^	4.62^a^	5.38^a^	7.31^a^	8.02^a^	28.8^a^	24.8^a^
Nutrient Expert + SPAD meter	400^a^	410^a^	59.44^a^	63.39^a^	41.4^a^	42.5^a^	4.72^a^	5.46^a^	7.46^a^	8.10^a^	29.4^a^	25.2^a^
SEm±	1.91	1.66	1.19	0.95	0.03	0.04	0.06	0.05	0.09	0.06	0.39	0.23
LSD (P = 0.05)	5.58	4.84	3.47	2.79	0.08	0.10	0.18	0.15	0.26	0.16	1.12	0.68

Treatment means with similar superscripted letters within a column are not significantly different at P < 0.05 according to Tukey’s honestly significant difference test.

Irrigation at 25% depletion of available soil moisture (DASM) outperformed both 50% DASM and 75% DASM + silicon (Si_80_) applications. This regime produced the highest effective tiller density, grains spike^-1^, and 1000-grain weight with statistically significant advantages. Grain yields under 25% DASM (5.39–6.04 t ha^−1^) surpassed 50% DASM by 10.91–7.67% and 75% DASM + Si80 by 50.98–39.81%, while straw yields (8.56–9.14 t ha^−1^) were also markedly higher.

Crops managed using nitrogen application guided by the NutrientExpert (NE) + SPAD meter exhibited superior tiller density (400–410 m^−2^), exceeding RDN by 7.82–8.18% and NE + leaf color chart (LCC) by 2.83–2.76%. Spike traits (grains spike-^1^: 59.44–63.40; 1000-grain weight: 41.40–42.50 g) were all superior under NE+SPAD. Grain (4.72–5.46 t ha^−1^) and straw yields (7.46–8.10 t ha^−1^) under this method exceeded RDN by 5.59–6.64% and 5.36–3.7%, respectively, though NE+SPAD and NE+LCC showed statistically comparable results (**Table 7**).

### 3.6 Partial factor productivity of N, P and K of wheat

Double zero-tillage wheat exhibited superior nutrient-use efficiency, with higher PFP_N_ (39.6–45.5 kg grain kg^−1^N), PFP_P_ (113.6–130.4 kg grain kg^−1^P), and PFP_K_ (119.2–136.9 kg grain kg^−1^K) compared to conventional ZT-wheat (PFP_N_: 36.9–42.9; PFP_P_: 105.7–123.0; PFP_K_: 110.0–129.2 kg grain kg^−1^) during both study years. Irrigation at 25% depletion of available soil moisture (DASM) resulted in the highest PFP_N_ (44.8–50.4), PFP_P_ (128.2–143.8), and PFP_K_ (134.7–151.0 kg grain kg^−1^), significantly surpassing 50% DASM and 75% DASM+Si_80_. Among precision nitrogen strategies, the NutrientExpert (NE) + SPAD meter approach yielded the highest PFP_N_ (42.9–49.6), PFP_P_ (112.5–130.0), and PFP_K_ (118.1–136.5 kg grain kg^−1^), significantly outwitting the RDN, and remaining statistically comparable to NE + LCC (**Table 8**).

**Table 8:** Effect of crop establishment methods, irrigation regimes and precision N management options on partial factor productivity of N (PFP_N_), P (PFP_P_) and K (PFP_K_) in wheat.

Treatments	PFP_N_ (kg grain kg^-1^ N applied)		PFP_P_ (kg grain kg^-1^ P applied)		PFP_K_ (kg grain kg^-1^ K applied)	
	2019-20	2020-21	2019-20	2020-21	2019-20	2020-21
Crop establishment methods						
Zero till-wheat	36.9^b^	42.9^b^	105.7^b^	123.0^b^	111.0^b^	129.2^b^
Double zero till-wheat	39.6^a^	45.5^a^	113.6^a^	130.4^a^	119.2^a^	136.9^a^
SEm±	0.69	0.70	1.99	2.12	2.09	2.23
LSD (P = 0.05)	2.16	2.21	6.26	6.68	6.58	7.00
Irrigation regimes						
Irrigation at 25% DASM	44.8^a^	50.4^a^	128.2^a^	143.8^a^	134.7^a^	151.0^a^
Irrigation at 50% DASM	40.4^b^	46.5^b^	115.7^b^	133.5^b^	121.5^b^	140.2^b^
Irrigation at 75% DASM +Si(80 kg ha^-1^)	29.6^c^	35.8^c^	85.0^c^	102.8^c^	89.2^c^	107.9^c^
SEm±	0.84	0.86	2.44	2.60	2.56	2.73
LSD (P = 0.05)	2.65	2.71	6.67	8.18	8.06	8.59
N management options						
Recommended N rate	29.8^b^	34.2^b^	106.3^b^	122.0^b^	111.7^b^	128.1^b^
Nutrient Expert + LCC	42.1^a^	48.9^a^	110.1^a^	128.1^a^	115.6^a^	134.5^a^
Nutrient Expert + SPAD meter	42.9^a^	49.6^a^	112.5^a^	130.0^a^	118.1^a^	136.5^a^
SEm±	0.55	0.38	1.46	1.25	1.54	1.32
LSD (P = 0.05)	1.61	1.10	4.27	3.66	4.48	3.84

Treatment means with similar superscripted letters within a column are not significantly different at P < 0.05 according to Tukey’s honestly significant difference test.

### 3.7 Irrigation water productivity

Irrigation water productivity (IWP) (29.8 and 25.2 kg ha^-1^ mm^-1^) was significantly superior under DZT-wheat over ZT-wheat (29.8 and 25.2 kg ha^-1^ mm^-1^, respectively) during both years (**Table 7**). IWP among different irrigation regimes ranged between 24.0 – 29.7 kg ha^-1^ mm^-1^, maximum being with irrigation at 75% DASM+ Si_80_ (29.7 kg ha^-1^ mm^-1^) and the minimum being with irrigation at 25% DASM (24 kg ha^-1^ mm^-1^). Among different precision N-management options, NE+ SPAD meter resulted in significantly higher IWP over RDN.

### 3.8 N_2_O emission

**Figure 2:**
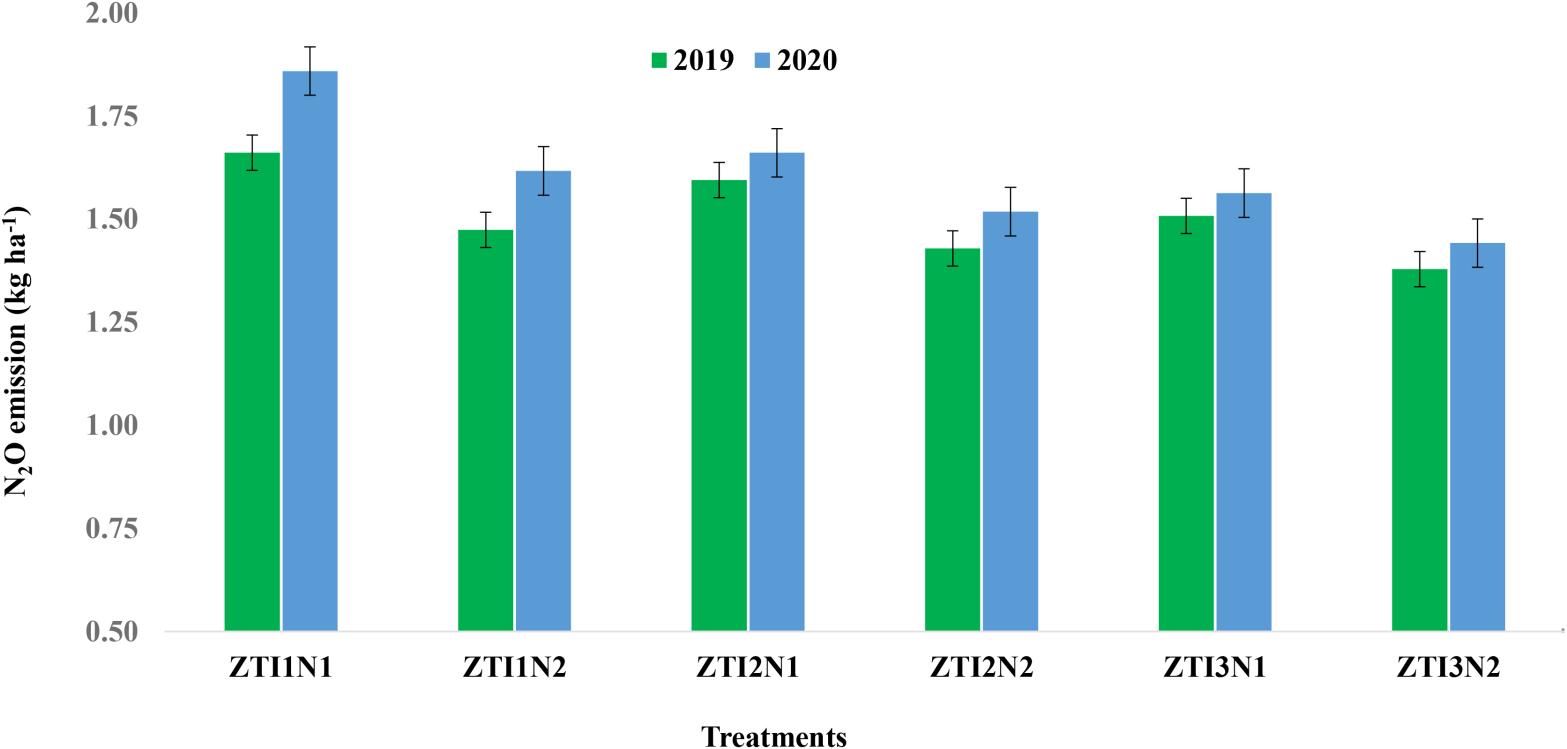
Effect of irrigation regimes and precision N management options on N_2_O emission in zero-till (ZT) wheat. Error bar indicate LSD (P<0.05). I1: 25% DASM; I2: Irrigation at 50% DASM; I3: Irrigation at 75% DASM + Si_80_; NI: Recommended N rate at 150 kg ha^-1^, N2: Nutrient Expert + LCC, ZT: Zero-tilled wheat.

A significant (P<0.05) difference on N_2_O emission in wheat was observed due to crop establishment methods, irrigation regimes and precision N-management options during both years (**Figure 2**). In general, a higher N_2_O emission was noticed during second year as compared to first year of the study. Combination of ZT × irrigation at 25% DASM × RDN emitted significantly higher N_2_O over all other combinations of ZT × irrigation regimes × N schedules. However, combination of ZT × irrigation at 75% DASM+ Si_80_ × NE+ LCC registered significantly lower N_2_O emission.

### 3.9 Pearson’s correlation analysis

**Figure 3:**
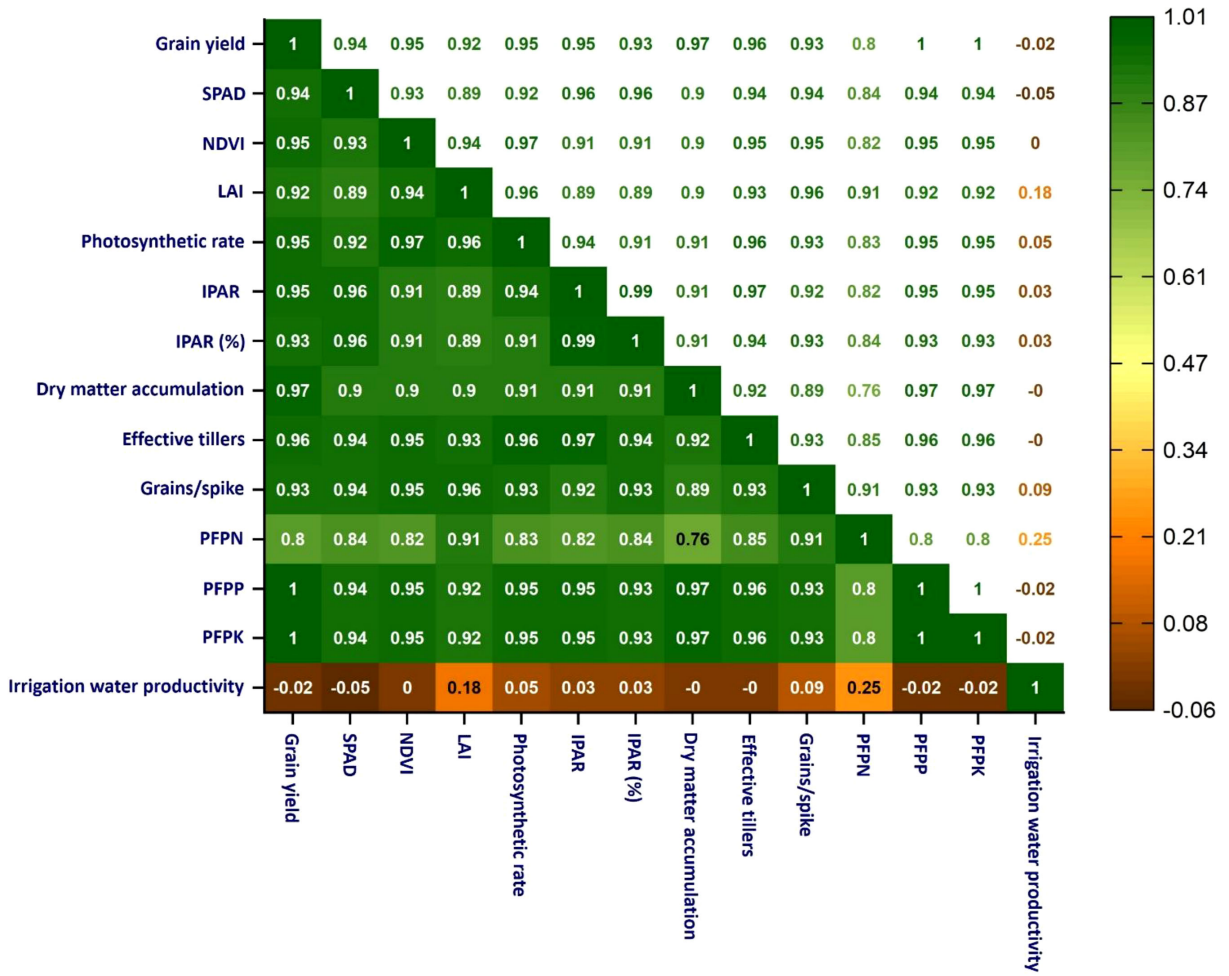
Pearson correlation matrix analysis of different growth and yield parameters. SPAD, Soil Plant Analysis Development; NDVI, Normalized Difference Vegetation Index; LAI, Leaf Area Index; IPAR, Intercepted Photosynthetically Active Radiation; IPAR (%), Percentage of intercepted photosynthetically active radiation; PFP, Partial Factor Productivity; PFP_N_, Partial factor productivity of nitrogen (kg grain per kg N applied); PFP_P_, Partial factor productivity of phosphorus (kg grain per kg P applied); PFP_K_, Partial factor productivity of potassium (kg grain per kg K applied). Correlation coefficients shown are significant at *p* < 0.05 and p < 0.01 unless otherwise stated.

Wheat grain yield showed strong positive correlations (**Figure 3**) with key physiological and vegetative metrics across both growing seasons, including SPAD (r = 0.94), NDVI (r = 0.95), LAI (r = 0.92), photosynthetic rate (r = 0.95), IPAR (r = 0.94), and DMA (r = 0.97).

## 4 Discussion

### 4.1 Growth parameters

Double ZT wheat cultivation recorded significantly higher DMA and LAI than conventional ZT systems. These improvements were attributed to the synergistic effects of residue retention and ZT practices, which regulate soil temperature (Varatharajan et al., 2019), enhance water infiltration and moisture conservation (Zribi et al., 2015), improve soil structure, suppress weed proliferation (Dass et al., 2017; Pratap et al., 2021a, Pratap et al., 2023), and enhance soil physico-chemical and biological health. Higher LAI under DZT is particularly important, as it enhances the interception of photosynthetically active radiation (PAR), which directly contributes to dry matter production and yield potential (Lopes et al., 2011). Optimized soil porosity under these conditions facilitated robust root development, improving nutrient and water uptake efficiency, which collectively enhanced vegetative growth and biomass production.

Optimal soil moisture under 25% DASM promoted tiller formation, leaf expansion, and sustained metabolic activity (Pratap et al., 2022). In contrast, the 75% DASM + Si_80_ treatment restricted growth due to moisture stress as irrigations were delayed till 75% of available moisture had been exhausted, and Si application not being able to sufficiently impart stress tolerance, reducing DMA and LAI (Pratap et al., 2022). Higher LAI under 25% DASM compared with 75% DASM + Si_80_ indicates that moisture availability plays a more decisive role than supplemental stress-alleviating inputs like silicon under severe water deficit (Salem et al., 2021b). Enhanced DMA under 25% DASM irrigation compared with 75% DASM + Si_80_ corroborates earlier findings that adequate soil moisture improves nutrient absorption, photosynthetic activity, and assimilate partitioning in wheat (Liang et al., 2019b). Additionally, NE-guided N application coupled with SPAD meter or LCC protocols, significantly increased crop growth, improving N uptake efficiency, root development, and leaf expansion (Mondal et al., 2018). The superiority of precision N management tools (NE + SPAD) over RDN is consistent with earlier reports showing that real-time crop-based diagnostic tools improve N-use efficiency and crop growth (Cui et al., 2018). As SSNM helps meet crop requirements and enhance soil fertility, it performed better than uniform fertilizer applications. SSNM was found to enhance DMA and LAI by addressing spatial and temporal variability in nutrient availability (Pratap et al., 2021b), thus promoting sustainable yield.

### 4.2 NDVI value

Higher NDVI values recorded under DZT-wheat were attributed to higher leaf N content resulting from higher N uptake, whereas lesser N content under ZT-wheat caused lower NDVI values (Singh et al., 2018). Our findings reveal that irrigation practices greatly affect NDVI measurements, showing a strong positive correlation with both biomass production and grain yield. Enhanced soil moisture retention and better root proliferation in DZT likely contributed to improved biomass accumulation and chlorophyll activity, as supported by earlier studies under conservation agriculture (Sapkota et al., 2021a). Higher NDVI values in wheat at 25% DASM over other irrigation regimes could be possible due to greater leaf N content, owing to higher N uptake resulting from greater solubilization of both applied and native nutrients under ideal soil moisture conditions. Lowest NDVI values under irrigation at 75% DASM + Si_80_ were attributed to lower leaf N content resulting from lesser N availability and mobility in the soil-plant continuum due to a lack of sufficient moisture (Irakli et al., 2011). Irrigation scheduling at 25% depletion of available soil moisture (DASM) significantly enhanced NDVI compared to 75% DASM + Si_80_, with 50% DASM showing intermediate results. This pattern indicates that adequate soil moisture supply during critical stages sustains photosynthetic activity and leaf area, while deficit irrigation reduces canopy greenness (Liang et al., 2019b; Lopes et al., 2011). The higher NDVI under optimum irrigation aligns with findings that soil moisture directly influences nitrogen uptake and chlorophyll expression (Hanjra et al., 2009). Higher NDVI values under both the NE-guided N application were primarily due to higher leaf N content because of greater N uptake by plants as imparted by the combined effect of balanced nutrient application at the sowing time of the crop and further need-based N supply (Li et al., 2013). Among precision N-management approaches, NE + SPAD consistently outperformed RDN, and was statistically comparable with NE + LCC. SPAD-based N application ensures real-time crop N status assessment, leading to better synchronization of N supply with crop demand, thereby maintaining higher NDVI (Cui et al., 2018). The lower NDVI values under RDN were attributed to lower leaf N content, due to inefficient uptake from a supply-demand mismatch caused by N loss when the application schedule did not align with the crop’s dynamic nitrogen demand.

### 4.3 Photosynthetic behaviors

Higher photosynthetic rate, stomatal conductance, and transpiration rate at both the study stages were found in DZT-wheat, attributed to higher chlorophyll content resulting from higher leaf N content consequent to greater N uptake by the plant (Singh et al., 2018). A similar finding was also reported by Thomas et al. (2005), they highlighted that chlorophyll content is crucial for achieving a higher photosynthetic rate and found a significant positive correlation between photosynthetic rate and SPAD values. Further, higher photosynthetic efficiency under ZT-wheat was primarily due to a lower photosynthetic rate. Adequate soil moisture regimes enhance leaf water potential, boosting photosynthetic rates. Conversely, moisture stress limits photosynthesis by reducing leaf water potential and relative water content, negatively impacting plant growth and yield (Hassan, 2006). Similar results were reported by Thierfelder et al. (2018), who noted enhanced gas exchange traits in conservation tillage systems due to better soil aeration and moisture conservation.

The highest net photosynthetic rate (NPR), stomatal conductance, and transpiration rate in wheat with irrigation at 25% DASM were attributed to higher cell turgidity under adequate soil moisture regimes (Kumari et al., 2017). In contrast, the lowest rates at 75% DASM+Si_80_ were due to moisture stress, partially mitigated by Si, but still resulting in lower leaf water potential and reduced stomatal conductance, leading to partial stomatal closure and decreased NPR and transpiration. Our results align with Gao et al. (2011), who found that reduced soil water content under limited moisture conditions decreased NPR and transpiration due to impaired stomatal conductance and photochemical reactions, ultimately reducing grain weight. The relatively higher photosynthetic efficiency under 75% DASM + Si80 suggests that under moderate stress, plants tend to improve water-use efficiency through tighter stomatal regulation, a mechanism also described in wheat by Lobell et al. (2020) and Daryanto et al. (2017). Both the NE+SPAD meter and NE+LCC proved superior to RDN in terms of NPR, stomatal conductance, and transpiration rate. This was attributed to higher chlorophyll content resulting from a higher N uptake owing to regular need-based N supply (Li et al., 2013). Lower NPR, stomatal conductance, and transpiration rate under RDN were possibly due to lower chlorophyll content. Further, higher photosynthetic efficiency under RDN was ascribed to a lower photosynthetic rate. Such physiological trade-offs between carbon gain and water conservation under reduced N have also been observed in wheat and maize (Cui et al., 2018; Lobell et al., 2020).

### 4.4 Photosynthetic active radiation interception

Double ZT-wheat captured 4.1% and 3.6% more PAR during the maximum tillering and flowering stages, respectively, compared to conventional ZT-wheat. This improvement was linked to a higher LAI, driven by better crop growth, soil structure, water management, and nutrient absorption under the double ZT system (Harish et al., 2022). In contrast, ZT-wheat showed reduced PAR interception due to a lower LAI, resulting from sub-optimal growth. These results are in agreement with Dass and Bhattacharyya (2017), who found that residue retention improved chlorophyll content (SPAD values) and photosynthetic efficiency by enhancing soil moisture and nutrient availability. Similar findings were reported by Song et al. (2024), who demonstrated that residue retention in conservation agriculture created favorable soil microclimatic conditions—lower soil temperature and higher soil moisture—leading to improved green area index (GAI) and greater PAR capture at anthesis. Higher intercepted PAR under assured irrigation at both study stages was ascribed to a larger assimilating area contributed by more number of tillers owing to vigorous crop growth favored by higher nutrient uptake under ideal soil moisture regimes (Dass and Chandra, 2013; Kumari et al., 2017). Additionally, Kandel et al. (2019) highlighted that balanced water-use and higher N levels improve chlorophyll synthesis and NPR, leading to better LAI, PAR interception, and higher biomass and yield. Recent work on smart irrigation scheduling supports this observation, showing that Crop Water Stress Index (CWSI)-based irrigation enhances irrigation efficiency, prevents water stress, and sustains crop biomass by optimizing canopy function (Agricultural Water Management, 2024). The NE+SPAD meter-based N schedule in wheat intercepted ~ 2.0 and 4.8% higher PAR at the maximum tillering stage and 2.3 and 7.5% at the flowering stage over NE+ LCC and RDN, respectively. Higher PAR interception under the NE+ SPAD meter resulted from higher canopy development due to profuse crop growth and development, owing to higher nutrient uptake as a balanced amount of nutrients was supplied at the time of sowing as predicted by NE, and further, need-based N was supplied matching crop demand. SPAD meter-based N estimation has been validated as a reliable proxy for chlorophyll content and leaf N concentration (Rath et al., 2024), both of which are directly linked to higher radiation use efficiency (RUE) and light interception. Lower PAR interception with RDN was due to limited canopy development from reduced nutrient uptake, indicating that improper N application causes N stress (Saha et al., 2015).

### 4.5 Yield attributes and yields

Double ZT-wheat exhibited a 5.7% increase in effective tillers, along with improvements in grains spike^-1^ and 1000-grain weight, resulting in 6.7% and 5.1% higher grain and straw yields, respectively, compared to conventional ZT wheat. These improvements were attributed to superior root development, balanced nutrient availability, and optimized source-sink dynamics, as highlighted in long-term studies (Singh et al., 2017; Jat et al., 2019). Enhanced growth indicators, particularly LAI and DMA, contributed to increased photosynthetic efficiency and nutrient translocation during flowering. Residue retention further amplified these benefits by improving soil moisture, nutrient accessibility, and weed suppression (Saad et al., 2014; Parihar et al., 2017). The superiority of double zero-tillage (DZT) wheat over conventional ZT in effective tiller density, spike traits, and yield attributes aligns with earlier reports that DZT improves soil aggregation, root growth, and nutrient availability due to enhanced residue decomposition and better root–soil contact (Leharwan et al., 2023).

Applying water at 25% DASM resulted in the highest values for effective tillers, grains spike^-1^ and 1000-grain weight compared to 50% DASM and 75% DASM + Si_80_. Additionally, irrigation at 25% DASM led to a 9.3% higher grain yield and 13.0% higher straw yield compared to 50% DASM. Higher yield attributes and yield under irrigation in wheat at 25% DASM were ascribed to better crop growth and development, higher nutrient absorption, and photosynthetic accumulation that enabled hastened development of yield attributes that led to the formation of higher grain and straw yield (Meena et al., 2015). Similar results were reported by Kaur et al. (2022), who found that frequent irrigation scheduling enhanced spike traits and 1000-grain weight, translating into higher yield gains in wheat under water-limited conditions.

Among the precision N management options, NE + SPAD meter produced maximum effective tillers, spike-length, grains spike^-1^, spike-weight, 1000-grain weight, and grain and straw yield over NE+LCC and RDN. The enhancement in effective tillers, grain, and straw yield was ~ 2.8, 1.8, and 1.7% over NE+LCC and 8, 6.1, and 4.5%, respectively, over RDN. Higher yield attributes and yield improvements under NE-based site-specific nutrient management were attributed to the optimal nutrient supply aligned with crop demand and soil nutrient availability, as reported by Singh et al. (2017), and Pratap et al. (2022). The integration of DZT with residue cover, precision N management, and optimized water-use enhances soil microbial activity, nutrient availability, and overall crop performance. These practices facilitate native nutrient mineralization increase soil organic matter, improve structure, reduce erosion, and enhance moisture retention (Jat et al., 2018; Das et al., 2020). These results corroborate recent studies highlighting the advantages of SPAD-guided N application in optimizing nitrogen use efficiency (NUE), chlorophyll content, and yield stability (Rath et al., 2024). By improving soil health and resource efficiency, this approach strengthens agricultural sustainability and climate resilience while aligning with global sustainable development goals.

### 4.6 Partial factor productivity of N, P and K of wheat

On average, double ZT-wheat registered 6.7, 6.8, and 6.7% greater partial factor productivity of N (PFP_N_), P (PFP_P_), and K (PFP_K_) over ZT-wheat, majorly due to higher grain yield under the former. Enhanced PFP_N_, PFP_P_, and PFP_K_ under DZT can be attributed to improved root growth and better synchronization of nutrient release with crop demand, which has also been observed in conservation agriculture systems globally (Khanal et al., 2023). Within irrigation regimes, the highest PFP_N_, PFP_P_ and PFP_K_ at 25% DASM resulted from the highest grain yield. These findings are in line with results from the North China Plain, where moderate irrigation levels, in combination with optimized nitrogen application, enhanced both yield and PFP_N_ in wheat (Qin et al., 2024). Further, NE+ SPAD meter showed ~ 44.5, 6.2, and 6.2% higher PFP_N_, PFP_P_ and PFP_K_ over RDN. Likewise, NE+ LCC resulted in 42.1, 4.3 and 4.3% higher PFP_N_, PFP_P_ and PFP_K_ over RDN. Higher PFP_N_, PFP_P_ and PFP_K_ under NE-guided N application supplemented either with SPAD meter or LCC was ascribed to lesser but crop demand synchronized application of N, P and K fertilizer coupled with increased yield. Kumar et al. (2018) reported significantly higher partial factor productivity (PFP) under SSNM due to increased crop yield, whereas lower PFP_N_, PFP_P_, and PFP_K_ under RDN resulted from bulk nutrient application and reduced yield. NutrientExpert-based N management strategies enhanced nutrient-use efficiency and yield stability compared to blanket recommendations (Khanal et al., 2023). These results reinforce that precision nitrogen management tools are critical for maximizing input use efficiency, sustaining productivity, and minimizing environmental footprints.

### 4.7 Irrigation water productivity

Double ZT-wheat recorded 6.7% higher IWP compared to ZT-wheat. Higher IWP with DZT-wheat was mainly due to higher grain yield. Rajanna and Dhindwal (2019a) also found the highest WP, IWP, and heat-use efficiency under ZT-wheat. Similar findings have been reported in global studies, where conservation tillage improved water productivity and resource-use efficiency in wheat-based systems (Govaerts et al., 2018). Higher IWP under 75% DASM + Si_80_ resulted from more efficient water use due to less frequent irrigation (Zhao et al., 2020). Silicon has been shown to improve stomatal regulation, photosynthetic efficiency, and water-use efficiency under limited irrigation, thereby enhancing IWP (Kang et al., 2016; Gong and Chen, 2020). Irrigation at 25% DASM registered the lowest IWP because of higher irrigation water use. Our results corroborate with earlier studies, which suggest that frequent irrigation in wheat at 25% maximum allowable DASM creates adequate soil moisture regimes, thereby producing higher grain and straw yields and WUE over irrigating wheat at 50% maximum allowable DASM (Kaur et al., 2018), while Zhao et al. (2020) found that applying water in wheat at longer intervals coinciding with moisture-sensitive stages contributed higher IWP over frequent irrigation. Both precision N management options outperformed RDN in IWP, driven by higher grain yield under NE+SPAD and NE+LCC. International studies corroborate this, showing that SPAD-guided precision N management enhances both nitrogen-use efficiency and water productivity in cereals by synchronizing crop demand with nutrient and water availability (Zhao et al., 2021).

### 4.8 N_2_O emission

Optimized fertilizer management integrated with improved agronomic practices significantly reduces GHG emissions. Combining optimal N rates with slow-release fertilizers lowers N_2_O emissions compared to conventional methods (Liu et al., 2015). Elevated N_2_O emissions under practices like ZT with excessive irrigation (25% DASM) and recommended N (RDN) were due to enhanced microbial nitrification-denitrification, waterlogged soils, and crop-N mismatches (Pathak et al., 2002). Zero tillage often leads to greater stratification of soil organic matter and mineral N near the soil surface (Alvarez et al., 2012), and when combined with frequent irrigation, conditions become conducive for denitrification losses. Conversely, ZT with moderated irrigation (75% DASM), silicon amendment (Si_80_), and demand-driven N application (NE + LCC) improves soil aeration, N uptake, and reduces N losses (Wang et al., 2007). Reduced irrigation frequency under 75% DASM likely limited anaerobic microsites, thereby suppressing denitrification (Li et al., 2019). Additionally, silicon application (Si80) may have contributed indirectly by improving plant water status and enhancing nitrogen uptake efficiency (Gong and Chen, 2020), reducing surplus soil N available for gaseous losses.NE-guided fertilization cuts N use by 15–35%, boosts wheat yields by 4–8%, and reduces global warming potential by 2–20%, saving 5.24 Mt CO_2_ eq. annually (Sapkota et al., 2021b). These strategies align with sustainable agriculture goals by balancing productivity, resource efficiency, and GHG mitigation. Evidence from international studies supports that adaptive N management strategies, particularly LCC- and sensor-based approaches, can lower N_2_O emissions by 15–40% compared to conventional blanket fertilizer application while maintaining yields (Xia et al., 2020; Yang et al., 2022).

### 4.9 Pearson correlation analysis

High inter-correlations of SPAD, NDVI, LAI, NPR, IPAR, and DMA also suggest tightly interlinked effects on yield. In contrast, irrigation water productivity showed a weak or negligible correlation with most variables, such as grain yield (-0.02), suggesting that irrigation water productivity did not follow the grain yield trend; irrigation water productivity is often low with application of higher irrigation levels and vice-versa within a certain range of irrigation levels. Among the PFP measures, PFP_N_ showed a moderate correlation with grain yield (r=0.80), meaning that PFP_N_ plays a significant role. The PFP_P_ and PFP_K_ were highly correlated with grain yield, which suggests that these are important for wheat productivity. Effective tillers and grains/spikes also had strong positive correlations with grain yield (r= 0.96 and 0.93, respectively), which indicates their importance as key yield determinants. Pratap et al. (2022) emphasized the strong positive correlation between establishment method, precision N, and water management in influencing wheat grain yield. They found that NE-guided balanced nutrient application, which considers the soil’s indigenous nutrient supply, environmental conditions, and target yield, along with need-based N supply using SPAD meter/LCC, ensures a consistent N supply, leading to improved wheat productivity.

## 5 Conclusion

Precision fertilizer and water management combined with conservation agriculture offers a win-win approach to enhance agricultural and environmental results in IGP of India. Results revealed that wheat (variety HD 3086) can be successfully grown under double zero-tillage (ZT) systems without significant yield loss in the North-western plains. Water management strategies, such as irrigating wheat at 25% depletion of available soil moisture (DASM) optimize growth under sufficient water availability. In water-scarce conditions, delaying irrigation to 50% DASM can save one irrigation cycle, redistributing water to additional wheat fields. Nitrogen management using the NE^®^+SPAD-based method outperformed conventional recommended doses (RDN), improving growth, yield, and resource efficiency while reducing N_2_O emissions, saving approximately 40 kg ha^-1^ of nitrogen. This study has the potential to achieve Sustainable Development Goals (SDGs) like Zero Hunger, Clean Water and Sanitation, Life Below Water, Life on Land, Responsible Consumption and Production, and Climate Action. Moreover, it could be recommended that farmers should adopt precision nitrogen management with tailored irrigation practices to maximize yield, profitability, and resource efficiency while minimizing environmental impact. In water-limited areas, intermittent irrigation at 50% DASM along with precision nitrogen techniques can boost productivity and conserve water. Integrating precision agriculture under conservation tillage systems enhances photosynthetic efficiency, light interception, and overall wheat performance, supporting higher yields, farmer incomes, and environmental sustainability. This approach is essential for scaling climate-resilient wheat production in India’s resource-constrained regions.

## Data Availability

The raw data supporting the conclusions of this article will be made available by the authors, without undue reservation.
